# Navigating the Diagnostic Journey in Pediatric Gastroenterology: Decoding Recurrent Vomiting and Epigastric Pain in a Child with Glutaric Aciduria Type II

**DOI:** 10.3390/children11030285

**Published:** 2024-02-26

**Authors:** Ho-Poh Kek, Wan-Long Tsai, Pao-Chin Chiu, Wen-Harn Koh, Ching-Chung Tsai

**Affiliations:** 1 Department of Pediatrics, E-Da Hospital, I-Shou University, No. 1, Yi-Da Road, Yan-Chao District, Kaohsiung City 82445, Taiwan, R.O.C.; ed114204@edah.org.tw (H.-P.K.); ed114519@edah.org.tw (W.-L.T.); ed110026@edah.org.tw (W.-H.K.); 2 Department of Pediatrics, Kaohsiung Veterans General Hospital, No. 386, Dazhong 1st Road, Zuo-Ying District, Kaohsiung City 813414, Taiwan, R.O.C.; pcchiu@vghks.gov.tw; 3 School of Medicine for International Students, College of Medicine, I-Shou University, No. 8, Yi-Da Road, Yan-Chao District, Kaohsiung City 82445, Taiwan, R.O.C.

**Keywords:** glutaric aciduria type II, multiple acyl-CoA dehydrogenase deficiency, paroxysmal and recurrent vomiting, epigastric pain

## Abstract

Background: Glutaric aciduria type II (GA II), also known as multiple acyl-CoA dehydrogenase deficiency (MADD), is a rare autosomal recessive metabolic disorder with varied manifestations and onset ages. Case Report: This study presents a distinctive case of a 10-year-old girl who experienced episodic, intermittent vomiting and epigastric pain, particularly aggravated by high-fat and sweet foods. Despite inconclusive physical examinations and routine laboratory tests, and an initial suspicion of cyclic vomiting syndrome, the persistence of recurrent symptoms and metabolic abnormalities (metabolic acidosis and hypoglycemia) during her third hospital admission necessitated further investigation. Advanced diagnostic tests, including urinary organic acid analysis and genetic testing, identified heterozygous pathogenic variants in the ETFDH gene, confirming a diagnosis of GA IIc. The patient showed a positive response to a custom low-protein, low-fat diet supplemented with carnitine and riboflavin. Significance: This case emphasizes the diagnostic challenges associated with recurrent, nonspecific gastrointestinal symptoms in pediatric patients, particularly in differentiating between common gastrointestinal disorders and rare metabolic disorders like GA II. It highlights the importance of considering a broad differential diagnosis to enhance understanding and guide future medical approaches in similar cases.

## 1. Introduction

Glutaric aciduria type II (GA II), also known as multiple acyl-CoA dehydrogenase deficiency (MADD), is an autosomal recessive disorder affecting fatty acid, amino acid, and choline metabolism, occurring in only about 1 in 250,000 newborns [1]. This condition results from mutations in the ETFDH, ETFA, and ETFB genes, leading to deficiencies in the electron transport chain, particularly in electron transfer flavoprotein (ETF) or electron flavoprotein dehydrogenase (ETFDH) [2]. These deficiencies cause lipid accumulation in cells like cardiomyocytes, hepatocytes, and renal tubular cells [3]. The mutations in GA II result in different clinical subtypes. The neonatal forms (type a and b) are characterized by severe symptoms such as vomiting, hypoglycemia, acidosis, and a distinctive sweaty foot odor. These forms are often fatal. In contrast, the late-onset variant (type c) is generally milder and often presents with diverse manifestations and a variable age of onset [2,3].

This case report highlights the diagnostic complexity of GA II, especially when it presents with symptoms like sporadic and recurring vomiting along with epigastric pain. It serves as an illuminating example of how typical gastrointestinal symptoms can intertwine with a rare metabolic disorder, presenting a significant diagnostic endeavor for healthcare professionals.

## 2. Case Presentation

A previously healthy female patient, aged 10 years and 10 months old, was referred to and admitted to our hospital on 18 November 2020, following a 2-day history of epigastric pain. Additionally, she had experienced paroxysmal and recurrent vomiting accompanied by epigastric pain over the previous nine months, predominantly triggered by the consumption of high-fat, creamy, and sweet foods. Notably, she did not present with other symptoms such as headaches, fever, diarrhea, skin rash, joint pain, muscle weakness, or bloody stools. She was admitted on three separate occasions within another three-month period.

Her growth parameters included a body height of 134.5 cm, between the 3rd and 15th percentiles according to Taiwan’s growth chart, with a Z-score of −1.24. Her body weight is 23.5 kg, below the 3rd percentile, with a Z-score of −2.5 to −3, and her BMI is 13, also below the 3rd percentile, with a Z-score of −2.5 to −3 [4]. The physical examination of the patient revealed that her abdomen was soft and not distended. Her muscle power in all limbs was rated 5/5, indicating normal strength. She experienced tenderness specifically in the epigastric region. To determine a diagnosis, we performed a comprehensive set of laboratory tests. These included a complete blood count with differential count, a comprehensive metabolic panel (assessing serum glucose level, electrolytes, liver enzymes, and kidney function), venous blood gas analysis, lactate and ammonia levels, erythrocyte sedimentation rate, thyroid function tests (TSH, T4, Free T4), serum amylase and lipase levels, stool analysis for routine and infectious studies, food allergy testing, and autoimmune markers (C3, C4, ANA). Additionally, a bone age study was conducted due to her short stature. Notably, all these laboratory results did not indicate obviously specific abnormalities.

To enhance our understanding of the patient’s condition, a comprehensive endoscopic examination was undertaken. This procedure revealed the presence of multiple congestive plaques in the lower body of the stomach, alongside mucosal erosion. A biopsy was obtained for advanced investigation. The ensuing histopathological evaluation of the gastric mucosa demonstrated a mild infiltration of chronic inflammatory cells, accompanied by a dispersion of eosinophils, enumerated as 8–12 eosinophils per high-power field upon microscopic analysis.

During the patient’s second admission, we carried out detailed imaging studies. The computed tomography (CT) scan showed mild dilation of the intestinal loops but no signs of obstruction or mass. Further, both upper gastrointestinal and small bowel series indicated unobstructed passage of the contrast agent through the stomach and intestines, suggesting normal gastrointestinal functioning.

Given the presence of metabolic acidosis and hypoglycemia in diagnostic tests during the third admission, and considering these conditions were not observed during the first and second admissions, we proceeded with urinary organic acid analysis. This report identified elevated concentrations of specific acids, including 3-hydroxybutyric, methylmalonic, ethylmalonic, 2-hydroxyglutaric, and 4-hydroxyphenyllactic acids. Blood acylcarnitine analysis was also performed and it revealed elevated levels of C4–C18. These findings pointed towards a diagnosis of GA II, accompanied by ketosis. The course of diagnosis is summarized in Table 1.

To confirm the genetic basis of this diagnosis, sequencing by next-generation sequencing technology was undertaken, leading to the identification of two compound heterozygous pathogenic variants in the ETFDH gene, as shown in Table 2. These variants were instrumental in the manifestation of GA IIc. Subsequently, the patient responded positively to treatment and exhibited tolerance to a prescribed low-protein and low-fat diet without experiencing any complications. Coenzyme Q10 (20 mg three times a day) was used to improve the patient’s muscle pathology, while Carnitine (0.5 g four times a day) expedited glutaric acid metabolism to reduce toxin buildup. Vitamin B_2_ (100 mg three times a day) was given to boost enzyme efficiency in treating GA IIc. Furthermore, the patient has shown significant progress in growth and development, and the frequency of hospital admissions has significantly decreased compared to before.

## 3. Discussion

In evaluating a child with recurrent vomiting, clinicians should consider a broad spectrum of potential diagnoses beyond just gastrointestinal conditions like infection, inflammation, or structural issues. This assessment should include exploring possibilities such as food allergies or intolerances, neurological disorders (for example, epilepsy or posterior fossa lesions), endocrine problems like adrenal insufficiency, metabolic diseases like mitochondrial cytopathies, and psychological factors, particularly in cases lacking a clear organic cause [5,6,7,8,9]. Additionally, in cases of recurrent abdominal pain, the differential diagnosis is similarly wide-ranging. It should encompass functional pain syndromes, various gastrointestinal disorders, genitourinary issues, food intolerances, stress-related conditions, and even more serious conditions like abdominal malignancies [10,11].

In this case of recurrent paroxysmal vomiting and epigastralgia, cyclic vomiting syndrome (CVS) was ever suspected after ruling out organic diseases. However, before confirming a CVS diagnosis, it is important to consider the four aspects outlined by Gelfand et al.: determining whether the vomiting is cyclical or simply recurrent, assessing for neurological or developmental abnormalities between episodes, checking for evidence of cerebral lesions, and identifying triggers such as illness, fasting, or a diet high in fat or protein [9,12]. The history of this case is compatible with Gelfand’s fourth suggestion. Therefore, this stepwise approach exemplifies the role of a comprehensive diagnostic approach, integrating clinical, laboratory, endoscopic, imaging, and genetic data, is crucial in identifying rare metabolic mimics among extensive differentials [10,11].

This case highlights the significant variability in the initial symptoms of GA II, a rare metabolic disorder. Instead of the neuromuscular deficits typically associated with late-onset GA II, this patient presented with unusual symptoms, including recurrent vomiting and abdominal pain. Such atypical symptoms illustrate the wide range of clinical phenotypes that GA II can manifest. The ETFDH mutation identified in this case is likely responsible for a partial enzyme deficiency, leading to a comparatively milder clinical presentation. In stark contrast, a complete deficiency of ETFDH is often linked to a severe neonatal onset, marked by profound hypotonia, hypoglycemia, and significantly increased mortality risk [2,13].

In evaluating our patient for GA II and related metabolic disorders, we conducted essential metabolic screenings, including urine organic acid analysis, blood acylcarnitine profiling, and genetic testing. These tests are crucial for detecting metabolic abnormalities characteristic of such conditions. The screenings identified organic acids—such as glutaric acid, lactic acid, ethylmalonic acid, 3-hydroxyisovaleric acid, adipic acid, 2-hydroxybutyric acid, 2-hydroxyglutaric acid, and 2-hydroxyisocaproic acid—and revealed a distinctive pattern of plasma acylcarnitine elevations ranging from C4 to C18, which are instrumental in diagnosing the disease. Genetic analysis pinpointed the heterozygous missense mutations c.250G>A (p.Ala84Thr) and c.353G>T (p.Cys118Phe) in the ETFDH gene. The former mutation is frequently found in South China, hinting at a potential founder effect—a scenario where a small group of individuals contributes a particular genetic mutation to subsequent generations—in this region [14]. This genetic insight, combined with our patient’s unique clinical presentation characterized by symptoms provoked by high-fat or creamy foods rather than the classical muscle weakness associated with late-onset GA II, emphasizes the disease’s variability. This calls for a comprehensive diagnostic approach [15,16]. The range of clinical manifestations, including episodic nausea, vomiting, hypoglycemia, and sensitivity to dietary triggers, underlines the importance for clinicians to consider a broad spectrum of symptoms and triggers when diagnosing GA II [17,18].

Previous studies not only support our findings but also highlight the effectiveness of personalized treatments, such as riboflavin supplementation for specific genetic mutations. These studies underscore the significance of tailored management strategies in addressing the identified phenotype–genotype correlations [16,19,20,21].

Our approach combines comprehensive diagnostic testing with personalized care for patients exhibiting unexplained metabolic symptoms, crucial for managing diverse conditions like GA II. Integrating metabolic profiling and genetic testing is key, especially for atypical cases, helping to clarify complex genotype–phenotype interactions. This strategy not only supports accurate diagnoses but also guides the creation of tailored treatment plans, thus improving our handling of metabolic disorders and ensuring patients receive optimal, targeted care.

The management of GA II requires a comprehensive approach, emphasizing dietary modifications. A carbohydrate-rich, low-protein, and low-fat diet, supplemented with carnitine and riboflavin, is essential. Riboflavin is particularly important for stabilizing the misfolded ETFDH protein [22]. The regular monitoring of blood sugar, ketone bodies, organic acids, and liver and heart functions, along with assessing nutritional status and providing timely interventions during illness, are key to preventing metabolic crises [18,23].

## 4. Conclusions

This case underscores the importance of considering a broad range of diagnoses, including rare metabolic disorders like GA II, when faced with recurring nonspecific gastrointestinal symptoms in pediatrics.

## Figures and Tables

**Table 1:** Clinical overview of a pediatric gastrointestinal case. It summarizes hospital admissions, symptoms, tests, findings, diagnoses, treatments, and outcomes, highlighting the diagnostic journey and treatment response.

Timeline	Hospital Stay Duration	Main Symptoms	Diagnostic Tests Performed	Findings	Diagnosis at Discharge	Treatment Given	Treatment Response
The first admission	5 days	2 days of epigastric pain; she experienced epigastric pain when consuming oil-rich, creamy, or milk-containing foods in past 9 months.	CBC/DC, Na, K, Cl, AST, ALT, BUN, Cr, ESR, TSH, T4, free T4, stool routine and culture, IgE, MAST, venous gas, lactate, ammonia, IgA, IgG, IgM, urine routine, upper GI endoscopy, bone age study	Glucose: 113 mg/dL (normal range: 70–100 mg/dL); PH (venous): 7.41 (normal range: 7.32–7.43); ammonia: 55 μg/dL (normal range: 12–66 μg/dL); lactate: 3.50 mg/dL (normal range: 4.5–19.8 mg/dL); ESR: 14 mm/h (normal range: 0–20 mm/h)	1. Acute gastritis 2. Gastroesophageal reflux disease	Intravenous fluids, Biotase, Gascon, Famotidine	Clinical condition improved, discharged with outpatient follow-up
The second admission (17 days after the first admission)	6 days	Over 10 episodes of vomiting since 1 day before admission	CBC/DC, glucose, ALT, AST, total bilirubin, lactate, amylase, lipase, upper GI endoscopy with biopsies, EEG, upper GI series with small bowel follow through, abdominal computed tomography	Glucose: 85 mg/dL (normal range: 70–100 mg/dL); lactate: 5.52 mg/dL (normal range: 4.5–19.8 mg/dL); histology of antrum: mild infiltration of chronic inflammatory cells and scattered eosinophils (8–12 per high power field)	1. Acute gastritis 2. Gastroesophageal reflux disease 3. Cyclic vomiting syndrome cannot be ruled out completely	Intravenous fluids, Metoclopramide, Dexlansoprazole, Cyprohepatidine	Gradually improved, tolerated oral intake well, discharged with outpatient follow-up
The third admission (40 days after the second admission)	4 days	Episodic epigastric pain and vomiting 4–5 times since 1 day before admission, and over 10 times on admission day	CBC/DC, CRP, Na, K, glucose, venous gas, urine organic acid study	Glucose: 44 mg/dL (normal range: 70–100 mg/dL); Ph (venous): 7.26 (normal range: 7.32–7.43); WBC: 15,660/μL (normal range: 3500–11,000/μL)	1. Acute gastritis 2. Gastroesophageal reflux disease 3. Suspect cyclic vomiting syndrome 4. Metabolic acidosis 5. Hypoglycemia	Intravenous fluids, Metoclopramide, Dexlansoprazole	Vomiting improved, appetite improved, discharged
The fourth admission (26 days after the third admission)	4 days	Frequent vomiting episodes for 3 days	CBC/DC, CRP, glucose, Na, K, BUN, Cr, AST, ALT	Glucose: 73 (normal: 70–100 mg/dL); urine organic acid at the third admission: glutaric aciduria type II, accompanied by ketosis	Suspected glutaric aciduria type II	Intravenous fluids, Metoclopramide, Dexlansoprazolen, diet education	Vomiting episodes improved, tolerated oral intake, discharged
OPD after fourth admission			Genetic testing performed, blood acylcarnitine analysis	ETFDH gene mutation identified		Low-protein, low-fat diet; riboflavin and carnitine supplementation	Symptoms improved

CBC/DC: complete blood count/differential count, Na: Sodium, K: Potassium, Cl: Chloride, AST: Aspartate Aminotransferase, ALT: Alanine Aminotransferase, BUN: Blood Urea Nitrogen, Cr: Creatinine, ESR: Erythrocyte Sedimentation Rate, TSH: Thyroid Stimulating Hormone, T4: Thyroxine, Free T4: Free Thyroxine, IgE: Immunoglobulin E, MAST: Mast Cell Activation Syndrome Test, PH (venous): Venous Blood pH, EEG: Electroencephalogram, GI: Gastrointestinal, ETFDH: Electron transfer flavoprotein dehydrogenase.

**Table 2:** This table lists heterozygous variants in the ETFDH gene and their pathogenicity status, indicating their role in metabolic disease in this case.

Gene	Variants	Zygosity	ACMG Classification
ETFDH	Exon 3, c.250G>A (p.Ala84Thr)	Het	Pathogenic
ETFDH	Exon 3, c.353G>T (p.Cys118Phe)	Het	Likely pathogenic

ETFDH: Electron transfer flavoprotein dehydrogenase, Het: heterozygous.

## Data Availability

Data are contained within the article.
